# Dataset for life cycle assessment of pet bottle waste management options in Bauru, Brazil

**DOI:** 10.1016/j.dib.2020.106355

**Published:** 2020-09-28

**Authors:** Eduardo J.P. Martin, Deborah S.B.L. Oliveira, Luiza S.B.L. Oliveira, Barbara S. Bezerra

**Affiliations:** a Sao Paulo State University – UNESP, College of Engineering, Av. Eng. Luiz Edmundo C. Coube 14-01 - Vargem Limpa, Bauru, SP 17033-360, Brazil; b USF, University of South Florida, 4202 E. Fowler Avenue, Tampa, FL 33620, USA

**Keywords:** Polyethylene terephthalate (PET) waste, Recycling, Incineration, Landfilling, Life cycle assessment

## Abstract

This paper contains a dataset and spreadsheet with the inventory, calculations and results for the Life Cycle Assessment of PET (polyethylene terephthalate) bottle waste management options in Bauru, Brazil. Data for the Life Cycle Inventory (LCI) was collected *in situ* at sorting cooperatives, through interviews with municipal authorities, and through literature review. Data analysis was performed using the SimaPro v8.0 software for assessment of environmental impacts, using the ReCiPe midpoint hierarchical method. The data and results contained in the spreadsheet are divided as follows: worksheet 1 provides a title page with the reference to this article; worksheet 2 provides the impact values for the impact categories considered; worksheet 3 presents the impact values for all collection and transportation fleets; worksheets 4 to 7 provide the impacts for each process and each stage (operation, emissions, and construction) for the case where: waste PET is disposed of in a landfill and the treated sludge is composted (worksheet 4); waste PET is incinerated (worksheet 5); waste PET is sent to sorting cooperatives and the reject is landfilled (worksheet 6); waste PET is sent to sorting cooperatives and the reject is incinerated (worksheet 7); worksheets 8 to 16 contain the impacts for each stage and the total impact for scenarios 1 to 9, which are combinations of the processes presented in worksheets 4 to 7, and the collection and transportation calculations for each scenario; worksheet 17 presents a summary of the net impact and the impact for each stage for all scenarios and all impact categories considered; worksheet 18 contains the impacts for each process and each stage for the case where waste PET is disposed of in a landfill, and the treated sludge is landfilled. The data in this paper are suitable for waste management researchers and professionals.

## Specifications Table

**Table:** 

Subject	Environmental Science
Specific subject area	Waste Management and Disposal
Type of data	Table, Figure and Spreadsheet
How data were acquired	For the LCI (Life Cycle Inventory) data were acquired through content analysis of municipal solid waste plans from the city of Bauru, through interviews with the municipal authorities SEMMA (Municipal Secretary of Environment) and EMDURB (Municipal Development Agency of Bauru), and *in situ* at the sorting cooperative facility ASCAM. Other data used in the LCI are from literature sources.
Data format	Raw and analyzed data, and concise numerical data.
Parameters for data collection	The data collection period was the entire year of 2019. The total quantities of waste collected were obtained for each type of waste collection: EMBURD (Conventional and Selective), SEMMA (Ecopoints) and ASCAM (Association of Waste Pickers of the city). A reference flow of 1 t of PET bottle waste was used for determining the LCI for landfilling, recycling and incineration of waste PET and the collection and transportation distances.
Description of data collection	The raw data was collected from interviews at EMDURB and SEMMA (total volume of PET bottle waste from conventional and selective collection and the respective final disposal) and *in situ* at ASCAM (weighing of the collected material, gravimetric analysis of the household solid waste collected, and the processing capacity of sorting cooperatives). Other data for LCI were collected from literature review (Table S3). Those raw data were analyzed and treated to be used in the SimaPro v8.0 software.
Data source location	Bauru, State of Sao Paulo, Brazil.
Data accessibility	The data are presented with this article.
Related research article	This Data in Brief paper is being submitted concurrently with the article submitted to the Journal of Waste Management: Martin, E. J. P.; Oliveira, D. S. B. L.; Oliveira, L. S. B. L.; Bezerra, B. S. (2020). Life cycle comparative assessment of PET bottle waste management options: A case study for the city of Bauru, Brazil. (In Press)

## Value of the Data

• The data are important for identifying the environmental impacts of solid waste management regarding PET bottle waste, and can complement the general waste management literature.
• This dataset can aid public management professionals within the scope of improving solid waste management policies, planning and actions.
• This dataset can be used to compare different solid waste management programs and measure their sustainability (locally and regionally).
• This dataset is useful to help formulate governmental policies regarding new waste disposal programs from an environmental perspective.
• This dataset presents important insights to be used in conjunction with social and financial indicators to provide a comprehensive sustainability analysis.

## 1 Data Description

The dataset describes the characteristics of the transportation distances for the waste management structure in Bauru (Table S1) and the municipality's collection and transportation fleet (Table S2), gathered through field surveys conducted in 2019. Table S3 presents descriptively each of the elements used in the life cycle assessment inventory of PET bottle waste, showing in each item the reference used. Nine scenarios were considered: (1) current (base) scenario (96.4% of reference flow is sent to landfill, 3.6% is sent to sorting cooperatives; (2) 50% to sorting cooperatives, 50% to landfill; (3) 50% to sorting cooperatives, 50% to incineration; (4) 50% to landfill, 50% to incineration; (5) 100% to sorting cooperatives (keeping the current collection distribution); (6) 100% to landfill; (7) 100% to incineration, (8) 100% to sorting cooperatives (50% collected in Ecopoints, 50% collected by selective collection), (9) 100% to sorting cooperatives (75% collected in Ecopoints, 25% collected by selective collection) [1]. Figs. S1–S8 present the system boundary for the eight alternative scenarios proposed for PET bottle waste management. The system boundary for the base scenario is presented in Martin et al. [1]. Table S4 describes the results for the following environmental impact categories for each of the proposed scenarios for 1 t of PET bottle waste: Climate change; Ozone depletion; Terrestrial acidification; Freshwater eutrophication; Human toxicity; Terrestrial ecotoxicity and Freshwater ecotoxicity. The impacts for the other categories can be seen in the accompanying spreadsheet. The spreadsheet contains the following worksheets:

• Worksheet 1 (entitled “Intro”): this tab contains a title page with the reference to this article.
• Worksheet 2 (entitled “Simapro processes”): this tab contains the impact values for the impact categories considered in the ReCiPe midpoint method for each process used in the Simapro software. References are provided for the sources from which the inventory was taken, and a brief description of each process is included. When applicable, the modifications and adjustments made to the original inventories are mentioned. The inventory is summarized in Table S3.
• Worksheet 3 (entitled “C&T”): this tab contains the impact values for all collection and transportation fleets considered in this study.
• Worksheet 4 (entitled “Landfill PET – S to Comp”): this tab contains the impacts for each process and each stage (operation, emissions, and construction) for the case where waste PET is disposed of in a landfill, and the WWTP sludge is composted. This was assumed to represent the sludge disposal practice in Jundiaí, the city where the leachate from the landfill near Bauru is sent for treatment.
• Worksheet 5 (entitled “Incineration PET”): this tab contains the impacts for each process and each stage (operation, emissions, and construction) for the case where waste PET is incinerated.
• Worksheet 6 (entitled “Recycling PET – Wpet to L”): this tab contains the impacts for each process and each stage (operation, emissions, and construction) for the case where waste PET is sent to sorting cooperatives, and then the sorted waste PET is sent to the recycling industry. In this tab, the rejected waste PET from the sorting cooperatives in Bauru is landfilled, and the rejected waste PET from the recycling industry in other cities is also landfilled.
• Worksheet 7 (entitled “Recycling PET – Wpet to I”): this tab contains the impacts for each process and each stage (operation, emissions, and construction) for the case where waste PET is sent to sorting cooperatives, and then the sorted waste PET is sent to recycling facilities located in other cities. In this tab, the rejected waste PET from the sorting cooperatives in Bauru is incinerated, while the rejected waste PET from the recycling industry in other cities is landfilled.
• Worksheets 8 to 16 (entitled “S1”, “S2”, “S3”, “S4”, “S5”, “S6”, “S7”, “S8” and “S9”: these tabs contain the impacts for each stage (operation, emissions, construction, C&T) and the total impact for scenarios 1 to 9, respectively. They also contain the collection and transportation calculations for each scenario.
• Worksheet 17 (entitled “Summary”): this tab contains a summary of the net impact and the impact for each stage for all scenarios and all impact categories considered.
• Worksheet 18 (entitled “Landfill PET – S to L “Extra”): this tab contains the impacts for each process and each stage (operation, emissions, and construction) for the case where waste PET is disposed of in a landfill, and the WWTP sludge is landfilled. This was assumed to represent the sludge disposal practice in S. Carlos/Caieiras, the cities where the PET recycling facilities are located.

## 2 Experimental Design, Materials and Methods

### 2.1 Data collection

Data was collected in the city of Bauru, in the state of São Paulo, Brazil during 2019. In this year, 96,300 t of residential Municipal Solid Waste (MSW) were collected. The collection of MSW occurs in four ways: (1) conventional collection carried out by EMDURB; (2) selective collection of recyclable materials by EMDURB; (3) selective collection of recyclable materials carried out by ASCAM; and (4) through Ecopoints, specific locations in the city where the population can drop off some types of waste and from where waste is transported to an appropriate place for final disposal or reuse. The transportation of waste from the Ecopoints was carried out by SEMMA. Data collection was performed through interviews at EMBURD and SEMMA, and *in situ* at ASCAM. The amounts of MSW and percentage of PET bottle waste in the MSW, as well as collection distances, were obtained for each of these four collection methods.

### 2.2 PET bottle waste assessment model

The Life Cycle Assessment (LCA) methodology established by ISO 14040 (2006) is divided into four steps: definition of objectives and scope, inventory analysis, impact assessment and interpretation.

### 2.3 Objectives and scope

The objective was to assess the environmental impacts of nine different scenarios for the disposal of PET bottle waste generated in the city of Bauru, Brazil. The functional unit was 1 t of PET bottle waste, and the total PET bottle waste collected in the city of Bauru in 2019 was used to calculate the percentages of the reference flow per 1 t. The functional unit and the reference flow deal exclusively with PET bottle waste and exclude other types of PET waste.

The system boundary starts when PET bottle waste is discarded by residents and ends when it is recycled or disposed of (incinerated or landfilled). Also included in the system are the PET bottle waste collection and transportation system and replacement of fossil-based PET granules (by esterification of the ethylene glycol and terephthalic acid process), when applicable in the scenarios that contain recycling. The system boundaries for scenarios 2 to 9 are presented in Figs. S1–S8, respectively. The system boundary for the current scenario is presented in the related article by Martin et al. [1].

### 2.4 Inventory analysis

Primary and secondary data were used for the inventory. Some primary data were presented in Tables 1 and 2 of Martin et al. [1]. Primary data consists of: energy and inputs for sorting cooperatives, amount of PET waste collected and transportation distances. Secondary data, and their references, are summarized in Table S3.

### 2.5 Impact assessment and interpretation of LCA

The ReCiPe Worldwide Midpoint (Hierarchical) v1.09 method was used to evaluate the life cycle impacts for all nine scenarios considered. This method contains 18 impact categories. The SimaPro v8.0 software was used to compute the environmental impacts for each process shown in the “Simapro processes” worksheet in the spreadsheet in the Appendix. The references for the processes used are shown in Table S3. The calculations for the impact of each scenario are presented in detail in the worksheets entitled S1 to S9 of the spreadsheet in the Appendix. The impacts for seven selected categories in each of the nine scenarios evaluated are shown in Table S4. The complete impact outcomes for all 18 categories are presented in the spreadsheet in the Appendix, in the worksheet entitled “Summary”.

The interpretation of results and discussion for the seven categories shown in Table S4 are presented in Martin et al. [1].

## CRediT authorship contribution statement

**Eduardo J.P. Martin:** Conceptualization, Methodology, Writing - original draft. **Deborah S.B.L. Oliveira:** Conceptualization, Methodology, Validation, Writing - original draft, Writing - review & editing. **Luiza S.B.L. Oliveira:** Conceptualization, Methodology, Validation, Writing - original draft, Writing - review & editing. **Barbara S. Bezerra:** Conceptualization, Writing - original draft, Writing - review & editing.

## Declaration of Competing Interest

The authors declare that they have no known competing financial interests or personal relationships that could have appeared to influence the work reported in this paper.

## Appendix Supplementary materials

Image, application 1

Image, application 2

## Footnotes

Supplementary material associated with this article can be found, in the online version, at doi:10.1016/j.dib.2020.106355.
