# Supplementary material for: Dataset for life cycle assessment of pet bottle waste management options in Bauru, Brazil
Source: Data Brief. 2020 Sep 28;33:106355. doi: 10.1016/j.dib.2020.106355 (PMC7569285; doi:10.1016/j.dib.2020.106355)
Supplement: Supplementary file 1 [file mmc1.docx]

Table S1. Transportation distances for waste management structure in Bauru.

| Transport description | Distance travelled (km) | Reference |
| --- | --- | --- |
| EMDURB conventional collection + transport to landfill | 130 | EMDURB* |
| EMDURB selective collection + transport to cooperatives | 90 | EMDURB* |
| ASCAM collection + transport to cooperatives | 90 | ASCAM** |
| Ecopoint – SEMMA transport from Ecopoints to cooperatives | 44 | SEMMA* |
| PET sorting reject transportation from sorting cooperatives to landfill | 42 | Authors’ calculation |
| PET bales transportation from sorting cooperatives to recycling facility | 255 | Authors’ calculation |
| Leachate transportation from landfill to WWTP in Jundiaí | 350 | Authors’ calculation |
| Slags/residues transportation from incineration plant to landfill | 100 | Assumed distance to proposed incineration plant |
| Sludge transportation from WWTP to landfill in S. Carlos/Caieiras | 11 | Author’s calculation |

Obs.:* Data collected through on-site interviews. Interview questions concerned the average distance for each type of waste collection and transportation of reject. **Data collected *in situ*.

Table S2. Collection and transportation fleet in Bauru.

|  | EMDURB conventional^1^ | EMDURB selective^1^ | SEMMA/ASCAM^2^ |
| --- | --- | --- | --- |
| Total no. of trucks | 21 | 6 | 7 |
| Average capacity (t) | 18 | 16.8 | 18 |
| % EURO 1 (1992)^*^ | - | 16.7 | - |
| % EURO 2 (1995-1997) | - | - | - |
| % EURO 3 (1999-2000) | 19.0 | 16.7 | - |
| % EURO 4 (2005) | 47.6 | 16.6 | - |
| % EURO 5 (2008) | 14.3 | 50.0 | 71.4 |
| % EURO 6 (2012) | 19.0 | - | 28.6 |

^*^Year(s) in parentheses indicate model-year for trucks to which emissions standards apply. ^1^Data from Plano Municipal Saneamento Básico (2016). ^2^Data from Prefeitura de Bauru 2010 and 2020.

Table S3. Inventory list: Description of the processes in worksheet 2 “Simapro processes”.

| **Item** | **Comments** | **Reference** |
| --- | --- | --- |
| Waste preparation facility construction, global | - Used for the construction of a materials recycling facility. - Capacity: 5E8 t in 50 yrs. - No modifications were made to original inventory. | Kägi et al. (2017) |
| Municipal waste incineration facility construction, global | - Used for the construction of a municipal incineration facility. - Capacity 100,000 t/yr; 40 yrs. - No modifications were made to original inventory. | Doka (2007) |
| Sanitary landfill facility construction, global | - Used for the construction of a sanitary landfill facility. - Capacity: 1.8E6 t in 30 yrs (150 yrs aftercare). - No modifications made to original inventory. | Doka (2007) |
| Wastewater treatment facility construction, global | - Used for the construction of a wastewater treatment facility. - Capacity: 5E9 L/yr; 30 yrs. - No modifications were made to original inventory. | Doka (2007) |
| Sewer grid construction, global | - Used for the construction of a sewer grid line. - Capacity: 5E9 L/yr; 110 km. - No modifications were made to original inventory. | Doka (2007) |
| Polyethylene terephthalate production, granulate, bottle grade, global | - Used for the commercial production of PET from ethylene glycol and TPA. - Water sources were changed to BR geography. - Electricity generation was changed to hydroelectric, BR geography. - Heat generation was changed to co-generation, diesel, BR. | PlasticsEurope (2017) |
| Polyethylene terephthalate production, granulate, bottle grade, recycled, global | - Used for the production of recycled PET (food grade) from waste PET. - Same changes were made as to the above item. - Assumed all impacts of the process are due to recycled PET production (original process allocated 99.5% to PET, and 0.5% to PE). | Kägi et al. (2017) |

Table S3. Inventory list (Cont’d).

| **Item** | **Comments** | **Reference** |
| --- | --- | --- |
| Sorting of waste PET | - Used for the mechanical sorting of waste PET prior to recycling. - See Table 2 in Martin et al. (2020). | Primary data; Martin et al. (2020). |
| Treatment of wastewater, average, global | - Used for the treatment of wastewater (WW) generated in PET recycling facilities and landfill leachate. - Neglected emissions/burdens due to sludge spreading (which were presented separately in original inventory). - Neglected burdens from incineration of part of the sludge (which were presented separately in original inventory). - Original inventory included emissions due to incineration of part of the sludge (which were not presented separately from WWTP emissions, so they were subtracted using another inventory). - In the scenarios considered, sludge resulting from WWTP is either composted (landfill leachate in Bauru) or landfilled (wastewater/leachate in S. Carlos/Caieiras). - Heat and electricity generations were adapted to BR. | Doka (2008) |
| Treatment of waste polyethylene terephthalate, municipal incineration, global | - Used for the emissions/operational burdens from incineration of waste PET. - Original process provided “net” energy from incineration. - The gross energy generation was calculated separately (using PET LHV, and energy efficiencies given in the inventory). - Avoided energy was calculated separately by subtracting “net” energy from “gross” energy. - Electricity generation for facility used was assumed to come from PET combustion - Heat for facility used was assumed to come from PET combustion. - Excess heat not used elsewhere in the incineration plant was assumed to be converted into electricity on a vapor turbine with 45% efficiency. - Total excess electricity was assumed to replace electricity (hydroelectric, BR.) | Doka (2013) |

Table S3. Inventory list (Cont’d).

| **Item** | **Comments** | **Reference** |
| --- | --- | --- |
| Treatment of waste polyethylene terephthalate, sanitary landfill, global | - Used for the emissions/operational burdens due to landfilling of waste PET. - Original inventory considered that sludge resulting from the WWTP of landfill leachate was incinerated. - Neglected operational burdens due to incineration of sludge. Operational burdens due to landfilling of sludge were added separately in another process. - Emissions due to incineration of sludge were not given separately from landfill/WWTP emissions, so they were subtracted using another process. - Electricity generation for use in process was changed to hydroelectricity, BR. | Doka (2007) |
| Process-specific burdens, sanitary landfill, global | - Used for the operational burdens of landfilling of sludge (produced in the WWTP of landfill leachate). - Was added to the process “Treatment of waste polyethylene terephthalate, sanitary landfill, global”. - Electricity was changed to hydroelectric, BR. - Heat for landfill operation came from LFG; excess heat is not recovered. | Doka (2007) |
| Treatment of digester sludge, municipal incineration, future, global | - Used for the emissions resulting from the incineration of digester (fermented) sludge resulting from WW treatment. - Was subtracted from the processes “Treatment of average wastewater, global” and “Treatment of waste PET, sanitary landfill, global”. - Electricity generation for facility used was adapted for BR. - Heat for facility used was assumed to come from PET combustion. | Jungbluth et al. (2007) |

Table S3. Inventory list (Cont’d).

| **Item** | **Comments** | **Reference** |
| --- | --- | --- |
| Treatment of municipal solid waste, sanitary landfill, global | - Used for the emissions resulting from the landfilling of sludge (produced in WWTP of landfill leachate). - Landfilling of sludge was approximated as landfilling of average MSW, since there were no specific processes for landfilling of sludge. - Landfilling of sludge is a common practice in the closed landfill in Bauru and in S. Carlos. - Was added to the process “Treatment of waste PET, sanitary landfill, global” | Doka (2007) |
| Composting facility construction, open, global | - Used for the construction of an open composting facility. - Capacity: 10,000 t/yr; 25 yrs. - No modifications were made to original inventory. | Nemecek et al. (2007) |
| Sewage sludge composting, operation | - Used for the operational burdens for the windrow (open) composting of sewage sludge. - Electricity was changed to hydroelectric, BR. | Cadena et al. (2009) |
| Sewage sludge composting, emissions | - Used for the direct emissions due to windrow (open) composting of sewage sludge. - Indirect emissions were excluded, since they were added using the inventory in the previous item. | Amlinger et al. (2008) |
| Transport, freight, lorry, 16-32 t. global | - Used for the emissions and operational burdens (such as road construction and maintenance, lorry construction and maintenance, indirect emissions due to tire, brake, and road wear) of transport freight in 16-32 t lorries. - Use of weed-killing chemicals (glyphosate) and defrosting agents were neglected. | Keller (2010) |

Landfill1

Transport

WWTP1

Composting

C&T

C&T

C&T

C&T

Coops.

Transport

Transport

Recycling (91% eff.)

Sewer grid

WWTP2

Landfill2

1 t waste PET

50%

EMDURB con.

28.5%

EMDURB sel.

5.0%

ASCAM

16.5%

Ecopoint - SEMMA

130 km

90 km

90 km

44 km

42 km

Reject (19.6%)

Sorted (80.4%)

255 km

350 km

Transport

42 km

Reject (9%)

Leachate

Leachate

Sludge

Sludge

Waste

water

0.37 t PET granulate

Fuel

Materials

Energy

Direct emissions

Indirect emissions

**Process data:**

0.071 L leachate/kg PET landfilled

1.07E-4 kg sludge/kg PET landfilled

6.37E-3 m^3^ WW/kg granulate PET

0.509 kg sludge/m^3^ WW

0.0232 L leachate/kg sludge landfilled

11 km

WWTP: Wastewater Treatment Plant

C&T: Collection and Transportation

Coops: Sorting Cooperatives

Figure S1. System boundary for scenario 2 (50% landfilled, 50% sent to sorting cooperatives, reject is landfilled).

Incineration

Transport

Landfill1

C&T

C&T

C&T

C&T

Coops.

Transport

Transport

Recycling (91% eff.)

Sewer grid

WWTP2

Landfill2

1 t waste PET

50%

EMDURB con.

28.5%

EMDURB sel.

5.0%

ASCAM

16.5%

Ecopoint - SEMMA

130 km

90 km

90 km

44 km

42 km

Reject (19.6%)

Sorted (80.4%)

255 km

100 km

Transport

42 km

Reject (9%)

Slags/ residues

Leachate

Sludge

Waste

water

0.37 t PET granulate

Fuel

Materials

Energy

Direct emissions

Indirect emissions

**Process data:**

1.9E-2 kg slags & residues/kg PET incinerated

6.37E-3 m^3^ WW/kg granulate PET

0.509 kg sludge/m^3^ WW

0.0232 L leachate/kg sludge landfilled

11 km

WWTP: Wastewater Treatment Plant

C&T: Collection and Transportation

Coops: Sorting Cooperatives

Figure S2. System boundary for scenario 3 (50% incinerated, 50% sent to sorting cooperatives, reject from cooperatives in Bauru is incinerated, reject from recycling facilities in S. Carlos/Caieiras is landfilled).

Landfill1

Transport

WWTP1

Composting

C&T

C&T

Incineration

1 t waste PET

50%

EMDURB con.

50%

EMDURB con.

130 km

130 km

350 km

Leachate

Sludge

Fuel

Materials

Energy

Direct emissions

Indirect emissions

**Process data:**

0.071 L leachate/kg PET landfilled

1.07E-4 kg sludge/kg PET landfilled

1.9E-2 kg slags & residues/kg PET incinerated

Transport

100 km

Slags/ residues

WWTP: Wastewater Treatment Plant

C&T: Collection and Transportation

Figure S3. System boundary for scenario 4 (50% landfilled, 50% incinerated).

Landfill1

Transport

WWTP1

Composting

C&T

C&T

C&T

Coops.

Transport

Transport

Recycling (91% eff.)

Sewer grid

WWTP2

Landfill2

1 t waste PET

57.1%

EMDURB sel.

9.9%

ASCAM

33.0%

Ecopoint - SEMMA

90 km

90 km

44 km

42 km

Reject (19.6%)

Sorted (80.4%)

255 km

350 km

Transport

42 km

Reject (9%)

Leachate

Leachate

Sludge

Sludge

Waste

water

0.73 t PET granulate

Fuel

Materials

Energy

Direct emissions

Indirect emissions

**Process data:**

0.071 L leachate/kg PET landfilled

1.07E-4 kg sludge/kg PET landfilled

6.37E-3 m^3^ WW/kg granulate PET

0.509 kg sludge/m^3^ WW

0.0232 L leachate/kg sludge landfilled

11 km

WWTP: Wastewater Treatment Plant

C&T: Collection and Transportation

Coops: Sorting Cooperatives

Figure S4. System boundary for scenario 5 (100% sent to sorting cooperatives, reject is landfilled)

Landfill1

Transport

WWTP1

Composting

C&T

1 t waste PET

100%

EMDURB con.

130 km

350 km

Leachate

Sludge

Fuel

Materials

Energy

Direct emissions

Indirect emissions

**Process data:**

0.071 L leachate/kg PET landfilled

1.07E-4 kg sludge/kg PET landfilled

WWTP: Wastewater Treatment Plant

C&T: Collection and Transportation

Figure S5. System boundary for scenario 6 (100% landfilled).

Incineration

Transport

Landfill1

C&T

1 t waste PET

100%

EMDURB con.

130 km

100 km

Slags/

residues

Fuel

Materials

Energy

Direct emissions

Indirect emissions

**Process data:**

1.9E-2 kg slags & residues/ kg PET incinerated

C&T: Collection and Transportation

Figure S6. System boundary for scenario 7 (100% incinerated).

Landfill1

Transport

WWTP1

Composting

C&T

C&T

C&T

Coops.

Transport

Transport

Recycling (91% eff.)

Sewer grid

WWTP2

Landfill2

1 t waste PET

42.5%

EMDURB sel.

7.5%

ASCAM

50%

Ecopoint - SEMMA

90 km

90 km

44 km

42 km

Reject (19.6%)

Sorted (80.4%)

255 km

350 km

Transport

42 km

Reject (9%)

Leachate

Leachate

Sludge

Sludge

Waste

water

0.73 t PET granulate

Fuel

Materials

Energy

Direct emissions

Indirect emissions

**Process data:**

0.071 L leachate/kg PET landfilled

1.07E-4 kg sludge/kg PET landfilled

6.37E-3 m^3^ WW/kg granulate PET

0.509 kg sludge/m^3^ WW

0.0232 L leachate/kg sludge landfilled

11 km

WWTP: Wastewater Treatment Plant

C&T: Collection and Transportation

Coops: Sorting Cooperatives

Figure S7. System boundary for scenario 8 (100% sent to sorting cooperatives, 50% collected in Ecopoints, reject is landfilled).

Landfill1

Transport

WWTP1

Composting

C&T

C&T

C&T

Coops.

Transport

Transport

Recycling (91% eff.)

Sewer grid

WWTP2

Landfill2

1 t waste PET

21.3%

EMDURB sel.

3.7%

ASCAM

75%

Ecopoint - SEMMA

90 km

90 km

44 km

42 km

Reject (19.6%)

Sorted (80.4%)

255 km

350 km

Transport

42 km

Reject (9%)

Leachate

Leachate

Sludge

Sludge

Waste

water

0.73 t PET granulate

Fuel

Materials

Energy

Direct emissions

Indirect emissions

**Process data:**

0.071 L leachate/kg PET landfilled

1.07E-4 kg sludge/kg PET landfilled

6.37E-3 m^3^ WW/kg granulate PET

0.509 kg sludge/m^3^ WW

0.0232 L leachate/kg sludge landfilled

11 km

WWTP: Wastewater Treatment Plant

C&T: Collection and Transportation

Coops: Sorting Cooperatives

Figure S8. System boundary for scenario 9 (100% sent to sorting cooperatives, 75% collected in Ecopoints, reject is landfilled).

Table S4. Environmental impact results for all scenarios. Impact values are expressed per metric ton (t) waste PET.

| **Scena-rios** | **Categories** | **Climate change** | **Ozone depletion** | **Terrestrial acidification** | **Freshwater eutrophication** | **Human toxicity** | **Terrestrial ecotoxicity** | **Freshwater ecotoxicity** |
| --- | --- | --- | --- | --- | --- | --- | --- | --- |
|  | ***Unit*** | **kg CO_2_e** | **kg CFC-11e** | **kg SO_2_e** | **kg Pe** | **kg 1,4-DBe** | **kg 1,4-DBe** | **kg 1,4-DBe** |
| S1 | *Net* | 46.0 | 2.13E-6 | -4.88E-2 | -8.81E-3 | 178 | 4.51E-2 | 40.6 |
|  | *Op.* | 7.3 | 9.05E-7 | 5.08E-2 | 1.13E-3 | 1.7 | 1.63E-3 | 3.79E-2 |
|  | *Em.* | 66.1 | N/A | 1.72E-3 | 1.63E-4 | 189 | 3.95E-2 | 41.0 |
|  | *Const.* | 2.6 | 4.66E-7 | 1.65E-2 | 4.43E-4 | 0.8 | 1.26E-4 | 2.04E-2 |
|  | *C&T* | 26.6 | 1.85E-6 | 8.59E-2 | 1.59E-3 | 6.8 | 1.13E-2 | 1.14E-1 |
|  | *A.P.* | -56.7 | -1.09E-6 | -2.04E-1 | -1.21E-2 | -20.7 | -7.48E-3 | -5.82E-1 |
| S2 | *Net* | -494 | -3.23E-6 | -2.41 | -1.47E-1 | -125 | -5.15E-2 | 19.6 |
|  | *Op.* | 43.5 | 8.85E-6 | 2.76E-1 | 1.37E-2 | 21.5 | 1.01E-2 | 4.37E-1 |
|  | *Em.* | 44.3 | N/A | 6.26E-3 | 2.27E-3 | 124 | 2.58E-2 | 26.9 |
|  | *Const.* | 4.8 | 4.89E-7 | 3.43E-2 | 3.46E-3 | 7.2 | 6.59E-4 | 1.70E-1 |
|  | *C&T* | 37.4 | 2.59E-6 | 9.92E-2 | 2.24E-3 | 9.6 | 1.59E-2 | 1.60E-1 |
|  | *A.P.* | -788 | -1.52E-5 | -2.83 | -1.69E-1 | -287 | -1.04E-1 | -8.1 |
| S3 | *Net* | 494 | -3.68E-6 | -2.29 | -1.47E-1 | 27.9 | 5.24E-2 | 33.0 |
|  | *Op.* | 44.9 | 8.93E-6 | 2.73E-1 | 1.42E-2 | 22.5 | 1.03E-2 | 4.58E-1 |
|  | *Em.* | 1,230 | N/A | 1.62E-1 | 2.27E-3 | 278 | 1.31E-1 | 40.7 |
|  | *Const.* | 5.8 | 3.35E-7 | 3.53E-2 | 4.11E-3 | 7.9 | 7.27E-4 | 1.97E-1 |
|  | *C&T* | 35.1 | 2.43E-6 | 9.28E-2 | 2.11E-3 | 9.0 | 1.49E-2 | 1.50E-1 |
|  | *A.P.* | -822 | -1.54E-5 | -2.86 | -1.70E-1 | -289 | -1.04E-1 | -8.5 |
| S4 | *Net* | 1,060 | 2.17E-6 | 2.35E-1 | 1.65E-3 | 329 | 1.39E-1 | 53.5 |
|  | *Op.* | 5.7 | 3.62E-7 | 3.11E-2 | 5.57E-4 | 1.0 | 1.16E-3 | 2.51E-2 |
|  | *Em.* | 1,060 | N/A | 1.32E-1 | N/A | 323 | 1.28E-1 | 53.6 |
|  | *Const.* | 3.3 | 3.35E-7 | 1.60E-2 | 7.46E-4 | 0.9 | 1.41E-4 | 3.15E-2 |
|  | *C&T* | 23.9 | 1.66E-6 | 7.95E-2 | 1.43E-3 | 6.1 | 1.01E-2 | 1.02E-1 |
|  | *A.P.* | -28.7 | -1.87E-7 | -2.28E-2 | -1.09E-3 | -2.0 | -2.68E-4 | -3.51E-1 |
| S5 | *Net* | -1,420 | -9.01E-6 | -4.96 | -2.96E-1 | -451 | -1.56E-1 | -3.1 |
|  | *Op.* | 82.6 | 1.74E-5 | 5.19E-1 | 2.72E-2 | 42.9 | 1.92E-2 | 8.66E-1 |
|  | *Em.* | 20.9 | N/A | 1.12E-2 | 4.53E-3 | 53.2 | 1.09E-2 | 11.7 |
|  | *Const.* | 7.1 | 5.15E-7 | 5.35E-2 | 6.72E-3 | 14.1 | 1.23E-3 | 3.33E-1 |
|  | *C&T* | 49.1 | 3.39E-6 | 1.13E-1 | 2.95E-3 | 12.5 | 2.09E-2 | 2.10E-1 |
|  | *A.P.* | -1,580 | -3.03E-5 | -5.66 | -3.37E-1 | -574 | -2.08E-1 | -16.2 |

Table S4. Environmental impact results for all scenarios. Impact values are expressed per metric ton (t) waste PET (Cont’d).

| **Scena-rios** | **Categories** | **Climate change** | **Ozone depletion** | **Terrestrial acidification** | **Freshwater eutrophication** | **Human toxicity** | **Terrestrial ecotoxicity** | **Freshwater ecotoxicity** |
| --- | --- | --- | --- | --- | --- | --- | --- | --- |
|  | ***Unit*** | **kg CO_2_e** | **kg CFC-11e** | **kg SO_2_e** | **kg Pe** | **kg 1,4-DBe** | **kg 1,4-DBe** | **kg 1,4-DBe** |
| S6 | *Net* | 101 | 2.55E-6 | 1.35E-1 | 1.90E-3 | 201 | 5.26E-2 | 42.2 |
|  | *Op.* | 4.5 | 2.89E-7 | 3.33E-2 | 1.54E-4 | 0.2 | 9.79E-4 | 6.94E-3 |
|  | *Em.* | 67.8 | N/A | 1.36E-3 | N/A | 194 | 4.06E-2 | 42.1 |
|  | *Const.* | 2.4 | 4.64E-7 | 1.51E-2 | 2.09E-4 | 0.3 | 8.48E-5 | 8.81E-3 |
|  | *C&T* | 25.8 | 1.79E-6 | 8.48E-2 | 1.54E-3 | 6.6 | 1.09E-2 | 1.10E-1 |
|  | *A.P.* | N/A | N/A | N/A | N/A | N/A | N/A | N/A |
| S7 | *Net* | 2,030 | 1.80E-6 | 3.36E-1 | 1.39E-3 | 457 | 2.26E-1 | 64.7 |
|  | *Op.* | 6.8 | 4.35E-7 | 2.88E-2 | 9.60E-4 | 1.8 | 1.34E-3 | 4.33E-2 |
|  | *Em.* | 2,050 | N/A | 2.62E-1 | N/A | 452 | 2.16E-1 | 65.2 |
|  | *Const.* | 4.2 | 2.06E-7 | 1.68E-2 | 1.28E-3 | 1.4 | 1.98E-4 | 5.43E-2 |
|  | *C&T* | 21.9 | 1.53E-6 | 7.42E-2 | 1.31E-3 | 5.7 | 9.31E-3 | 9.37E-2 |
|  | *A.P.* | -57.3 | -3.71E-7 | -4.55E-2 | -2.17E-3 | -4.0 | -5.35E-4 | -7.02E-1 |
| S8 | *Net* | -1,420 | -9.10E-6 | -4.97 | -2.96E-1 | -452 | -1.56E-1 | -3.1 |
|  | *Op.* | 82.6 | 1.74E-5 | 5.19E-1 | 2.72E-2 | 42.9 | 1.92E-2 | 8.66E-1 |
|  | *Em.* | 20.9 | N/A | 1.12E-2 | 4.53E-3 | 53.2 | 1.09E-2 | 11.7 |
|  | *Const.* | 7.1 | 5.15E-7 | 5.35E-2 | 6.72E-3 | 14.1 | 1.23E-3 | 3.32E-1 |
|  | *C&T* | 47.7 | 3.30E-6 | 1.08E-1 | 2.87E-3 | 12.2 | 2.03E-2 | 2.05E-1 |
|  | *A.P.* | -1,580 | -3.03E-5 | -5.66 | -3.37E-1 | -574 | -2.08E-1 | -16.2 |
| S9 | *Net* | -1,420 | -9.24E-6 | -4.98 | -2.96E-1 | -452 | -1.57E-1 | -3.1 |
|  | *Op.* | 82.6 | 1.74E-5 | 5.19E-1 | 2.72E-2 | 42.9 | 1.92E-2 | 8.66E-1 |
|  | *Em.* | 20.9 | N/A | 1.12E-2 | 4.53E-3 | 53.2 | 1.09E-2 | 11.7 |
|  | *Const.* | 7.1 | 5.15E-7 | 5.35E-2 | 6.72E-3 | 14.1 | 1.23E-3 | 3.32E-1 |
|  | *C&T* | 45.8 | 3.17E-6 | 9.94E-2 | 2.75E-3 | 11.7 | 1.95E-2 | 1.97E-1 |
|  | *A.P.* | -1,580 | -3.03E-5 | -5.66 | -3.37E-1 | -574 | -2.08E-1 | -16.2 |

Source: Spreadsheet, worksheet “Summary”. Note: Op. stands for Operation; Em. for Emissions; Const. for Construction; and A. P. for Avoided Products.
